# The Effect of Psychological Dependence on Smoking Cessation †

**DOI:** 10.3390/healthcare14193172

**Published:** 2026-09-24

**Authors:** Yavuz Korkmaz, Adem Taha Özdemir

**Affiliations:** Department of Family Medicine, Malatya Turgut Özal University, 44210 Malatya, Türkiye; adem.ozdemir@ozal.edu.tr

**Keywords:** psychological dependence, nicotine dependence, smoking cessation, Fagerström test, withdrawal symptoms

## Abstract

**Background/Objectives**: This study aimed to examine the relationships between psychological dependence level and nicotine dependence, smoking cessation status, and withdrawal symptoms in smokers, as well as to identify the factors associated with nicotine dependence and smoking cessation status. **Methods**: This prospective cohort study included 513 participants who presented to a smoking cessation outpatient clinic. Data were collected using a sociodemographic data form, the Fagerström Test for Nicotine Dependence (FTND), and the Test to Assess the Psychological Dependence on Smoking (TAPDS). Smoking status was evaluated eight weeks after the completion of treatment. **Results**: The mean age of the participants was 36.7 ± 10.6 years, and 60.4% were male. A statistically significant association was found between psychological dependence level and nicotine dependence (*p* < 0.001). In the low psychological dependence group, low and very low nicotine dependence were observed more frequently than expected, while in the severe psychological dependence group, high nicotine dependence was observed more frequently than expected. A significant association was also found between psychological dependence and smoking cessation status; in the low psychological dependence group, those who quit smoking were observed more frequently than expected (60.0%), while in the severe psychological dependence group, those who could not quit were observed more frequently than expected (61.9%, standardized residual +5.90). Pearson correlation analysis revealed a moderate, positive, and significant relationship between psychological dependence and nicotine dependence (r = 0.41, *p* < 0.001). Multiple linear regression analysis determined that psychological dependence was the variable most strongly associated with nicotine dependence (β = 0.375, *p* < 0.001). Multinomial logistic regression analysis demonstrated that a one-unit increase in psychological dependence score was associated with lower odds of quitting smoking (OR = 0.920, 95% CI: 0.896–0.944, *p* < 0.001) and of reducing smoking (OR = 0.971, 95% CI: 0.944–0.998, *p* = 0.034). **Conclusions**: Psychological dependence was significantly associated with nicotine dependence and with lower smoking cessation success. The assessment of psychological dependence and the adoption of multidisciplinary approaches accordingly in smoking cessation treatment may increase treatment success.

## 1. Introduction

The use of tobacco products, including cigarettes, causes serious health problems and mortality worldwide and in Turkey. More than 7 million people die annually due to tobacco use, while millions more suffer from serious health conditions such as cardiovascular diseases, respiratory disorders, and more than 20 types of cancer [1]. The physical dependence effect of tobacco develops through nicotine. The addictive effect of nicotine arises from its direct impact on the brain’s reward system. During this process, the release balance of neurotransmitters such as dopamine and noradrenaline is altered, reinforcing repetitive consumption behavior in the user [2,3]. Physical dependence is defined as the compulsive use of nicotine, and the withdrawal syndrome that emerges following tobacco consumption is one of the most prominent physiological indicators of this dependence [4]. However, smoking dependence is not limited to physiological effects; the psychological dimension is at least as determinant as the physical dimension. In addition to the psychoactive properties of nicotine, the meanings attributed by the individual to the act of smoking also play an important role in maintaining dependence. For example, smoking may serve functions such as pleasure, social interaction, increased self-confidence, or status symbol for some individuals. Therefore, smoking dependence should be addressed with a multidimensional approach, and both biological and psychosocial factors should be evaluated together [5].

Psychological dependence is a type of dependence that develops against a substance or behavior, encompassing mental, emotional, and behavioral dimensions. Unlike physical dependence, psychological dependence is more concerned with how the individual feels, thinks, and behaves without the substance [6]. After the habit is established in tobacco use, the smoker begins to associate the act of smoking with a series of environmental stimuli. The conditions that trigger environmental stimuli may vary depending on the smoker’s environment. The most frequently encountered conditions may be associated with specific places such as waiting rooms, bars, restaurants, and inside cars while driving; emotional factors such as stress, anxiety, boredom, and loneliness; or social activities such as parties, business meetings, cocktails, and friend gatherings. For instance, the act of smoking becomes a habit adopted by the smoker, accompanied by various behaviors to obtain the daily nicotine dose. The smoking behavior itself, reinforcement of nicotine, environmental factors, the taste and smell of tobacco, and the act of holding a cigarette in hand become pleasant elements when they occur together over time. Consequently, these combined elements become associated with tobacco consumption by smokers [6,7].

Identifying the factors affecting success in the smoking cessation process is of great importance for developing effective intervention strategies. Although the literature has firmly established that the level of nicotine dependence influences smoking cessation success, most previous studies have focused on physical/nicotine dependence assessed with the Fagerström Test for Nicotine Dependence (FTND). Comparatively less attention has been paid to the independent predictive role of psychological dependence assessed with standardized, multidimensional instruments such as the Test to Assess the Psychological Dependence on Smoking (TAPDS), which captures distinct dimensions of dependence, including emotional regulation, image/self-reward, indifference, and social acceptance, particularly with respect to short- and medium-term cessation success and withdrawal symptoms. Psychological dependence may have significant effects on individuals’ motivation to quit smoking, the sustainability of quit attempts, and the risk of relapse. Furthermore, elucidating the relationship between psychological dependence and withdrawal symptoms may contribute to a better understanding of the difficulties experienced during the cessation process. In this context, comprehensive studies examining the relationship of psychological dependence with nicotine dependence, smoking cessation status, and withdrawal symptoms in individuals presenting to smoking cessation outpatient clinics in Turkey appear to be insufficient. This study aimed to examine the relationships between psychological dependence level and nicotine dependence, smoking cessation status, and withdrawal symptoms in smokers, as well as to identify the factors associated with nicotine dependence and smoking cessation status. The primary focus of this study was the relationship between psychological dependence and smoking cessation, whereas nicotine dependence and withdrawal symptoms were examined as secondary and complementary outcomes. Based on the above rationale, we tested the following hypotheses: (H1) psychological dependence is positively associated with, and an independent predictor of, nicotine dependence; (H2) higher psychological dependence is associated with lower odds of quitting and of reducing smoking; (H3) higher psychological dependence is associated with a greater likelihood of withdrawal symptoms; and (H4) psychological dependence predicts smoking cessation outcomes beyond nicotine dependence and sociodemographic characteristics. The proposed relationships among the study variables are summarized in the conceptual framework (Figure 1).

## 2. Materials and Methods

### 2.1. Study Design and Setting

This prospective cohort study was conducted between August 2025 and July 2026 on patients presenting to the Smoking Cessation Outpatient Clinic of Malatya Training and Research Hospital. The study was approved by the Health Sciences Scientific Research Ethics Committee of Malatya Turgut Özal University (protocol code 2025/261; meeting no. 6; date of approval: 30 July 2025) and was conducted in accordance with the Declaration of Helsinki. Written informed consent was obtained from all participants.

### 2.2. Study Population and Sample

The study population consisted of smokers who presented to the Smoking Cessation Outpatient Clinic of Malatya Training and Research Hospital. The sample size was determined using power analysis with G*Power 3.1 (Heinrich Heine University, Düsseldorf, Germany). Using the formula for an unknown population, a target of at least 384 participants was set with a 95% confidence interval, assuming a smoking dependence prevalence of 50%. Individuals aged 18 years and older who were in the smoking cessation process and agreed to participate in the study were included.

### 2.3. Exclusion Criteria

The following individuals were excluded from the study:

- Pregnant and breastfeeding individuals;
- Individuals under 18 years of age;
- Those with psychiatric disorders;
- Individuals with communication and language barriers.

### 2.4. Data Collection Instruments

Three main instruments were used for data collection in the study:

#### 2.4.1. Sociodemographic Information Form

A questionnaire form prepared by the researchers containing data on participants’ age, sex, education level, income status, presence of comorbidities, medication use, alcohol consumption, smoking history (pack-years), and knowledge level regarding passive/thirdhand smoke.

#### 2.4.2. Fagerström Test for Nicotine Dependence (FTND)

The FTND was developed to assess the level of physical dependence on nicotine. Developed by Karl Fagerström, this scale consists of 6 items and is scored out of a total of 10 points.

The Turkish version was first developed in 2004 by Uysal et al. [8] using a sample of 169 smokers; this study reported that the Turkish version had moderate reliability (Cronbach’s alpha: 0.56) and could be used as a measurement tool for assessing nicotine dependence in smoking cessation clinics. In later years, a more comprehensive validity and reliability study was conducted by Uysal and colleagues in Istanbul with 502 volunteers. In this study, Confirmatory Factor Analysis (CFA) confirmed that the scale had a two-factor structure; all items had factor loadings of 0.40 or above, and goodness-of-fit indices (CFI: 0.922, TLI: 0.902, RMSEA: 0.081) were found to be at adequate levels [8,9]. The scoring of the test is performed by summing the points determined according to the response to each question, with 0–2 score indicating “Very Low,” 3–4 score “Low,” 5–7 score “Moderate,” and 8–10 score “High” level of nicotine dependence. The six questions in the scale are: ‘how many cigarettes do you smoke daily’, ‘when do you smoke your first cigarette after waking up’, ‘do you find it difficult to refrain from smoking in prohibited areas’, ‘do you smoke even when confined to bed due to illness’, ‘do you smoke more frequently during the early hours of the day’, and ‘which cigarette is the hardest to give up’. Analyses revealed that the Turkish version of the scale has a two-factor structure. It has been shown that utilizing this two-factor structure in clinical practice allows for a more accurate assessment of patients’ nicotine dependence.

#### 2.4.3. Test to Assess the Psychological Dependence on Smoking (TAPDS)

The TAPDS was developed by Ponciano-Rodríguez et al. (2015) to evaluate the psychological components of smoking dependence using a multidimensional approach [4]. The study on the Turkish validity and reliability of this 25-item scale was conducted by Bardakci et al. (2021) [10]. The scale structurally consists of four subdimensions: Emotional Regulator (8 items), Image/Self-Reward (8 items), Indifference (5 items), and Social Acceptance (4 items). Scale items are scored using a three-point Likert-type rating system, with “Very Often” scored as 3 points, “Sometimes” as 2 points, and “Never” as 1 point. Dependence levels are classified based on the total score obtained: 25–41 points indicate mild dependence, 42–58 points indicate moderate dependence, and 59–75 points indicate severe dependence. The validity and reliability study of the Turkish version was conducted on data collected from 420 participants. In the Confirmatory Factor Analysis (CFA) conducted within the scope of validity analyses, all items had factor loadings above 0.30 and were statistically significant; goodness-of-fit indices (CFI: 0.95, TLI: 0.94, NFI: 0.93) revealed that the model had very good fit with the data. Within the scope of reliability analyses, Cronbach’s α and McDonald’s ω values calculated for the total score of the scale were 0.93, demonstrating that the scale has high reliability. When the reliability of subscales was examined, the Emotional Regulator, Image/Self-Reward, and Indifference subscales were found to have high reliability, while the Social Acceptance subscale was found to have moderate reliability. In light of all these findings, it was concluded that the Turkish version of the Test to Assess the Psychological Dependence on Smoking (TAPDS) is a sufficient, valid, and highly reliable measurement tool for assessing psychological dependence in smokers.

#### 2.4.4. Withdrawal Symptoms Assessment

The presence/absence of withdrawal symptoms during the smoking cessation process was recorded for each participant.

### 2.5. Data Collection Procedure

The study was planned prospectively. Participants who presented to the smoking cessation outpatient clinic and met the study criteria were informed about the study at their first visit, and written informed consent was obtained. At the initial interview, participants were administered the sociodemographic information form, FTND and TAPDS tests.

Within the scope of routine treatment protocols, participants were started on smoking cessation methods (nicotine gum, nicotine patch, cytisine, motivational interviewing, varenicline, bupropion, etc.). Treatment methods were determined by the clinician independently of the research protocol. Cytisine was administered as a standard 25-day course, whereas the other pharmacotherapies (e.g., nicotine gum, nicotine patch, varenicline, and bupropion) were applied for approximately two months; the treatment duration therefore differed according to the method used.

Smoking cessation status (quit, could not quit, reduced), withdrawal symptoms, and treatment methods were assessed eight weeks after the completion of the respective treatment course through outpatient clinic follow-up visits. Participants who did not attend follow-up visits were contacted by telephone to obtain information about their smoking cessation process, and the data form was completed. The study was terminated upon reaching the target number of participants or the end of the study period (July 2026).

### 2.6. Statistical Analysis

Jamovi software (version 2.6.44) was used for the statistical analysis of the data. Categorical variables were presented as counts and percentages [n (%)], while continuous variables were summarized as mean ± standard deviation or median (IQR), depending on the assumption of normality. The normality of continuous variables was assessed using the Shapiro–Wilk test. The *t*-test and one-way ANOVA were used to compare continuous variables with a normal distribution, whereas the Mann–Whitney U test and Kruskal–Wallis test were employed for data that did not follow a normal distribution. The Pearson chi-square test was used to examine relationships between categorical variables; Fisher’s exact test was applied when more than 20% of the expected frequencies were less than 5. In cases where the chi-square test yielded significant results, standardized residuals were examined to identify which groups differed, and values with |residual| > 1.96 were considered significant deviations. Pearson correlation analysis was performed to determine the direction and strength of the relationship between two continuous variables when the data followed a normal distribution, while Spearman correlation analysis was used when the data did not. Multiple linear regression analysis was conducted to identify factors associated with a continuous dependent variable (nicotine dependence). Age, pack-years, and psychological dependence were included as independent variables in the model. Regression model assumptions (linearity, multicollinearity, autocorrelation, and normal distribution of residuals) were examined; multicollinearity was checked using Variance Inflation Factor (VIF) and Tolerance values. In multiple linear regression analysis, estimated marginal means of nicotine dependence were calculated according to psychological dependence level, and comparisons were made at three different levels: one standard deviation below the mean, at the mean, and one standard deviation above the mean of psychological dependence score. Multinomial logistic regression analysis was performed to identify factors associated with a categorical dependent variable (smoking cessation status). The “Not Quit” group was used as the reference category, and the “Quit” and “Reduced” groups were compared relative to this group. Gender, education level, income level, nicotine dependence, and psychological dependence were included as independent variables in the model. Odds ratios (OR), 95% confidence intervals (95% CI), and regression coefficients (β) were presented for all analyses; model significance and goodness-of-fit were assessed. Tests with *p* < 0.05 were considered statistically significant. The predictors entered into the regression models were selected on theoretical and empirical grounds rather than solely on the basis of statistical significance in bivariate analyses. In the multiple linear regression predicting nicotine dependence, psychological dependence was specified as the focal predictor, while age and pack-years were included a priori as established determinants of nicotine dependence, representing demographic and cumulative-exposure influences, respectively. In the multinomial logistic regression predicting smoking cessation status, gender, education, and income were retained as sociodemographic covariates alongside nicotine and psychological dependence; age and pack-years were not entered into this model because they act primarily as determinants of nicotine dependence rather than as direct predictors of cessation, and because a more parsimonious specification was preferred to avoid overfitting given the three-category outcome.

## 3. Results

The demographic and clinical characteristics of the study population are presented in Table 1. The mean age was 36.7 years (SD = 10.6), and the sample was predominantly male (60.4%). The mean pack-year history was 24.7 (SD = 21.7), indicating moderate-to-heavy smoking exposure. Most participants were married (59.6%), university graduates (52.2%), and actively employed (62.6%). Regarding income, 54.8% reported income equal to expenses, while 26.7% had income exceeding expenses. Comorbidities were present in 22.6% of participants, medication use in 20.9%, and alcohol consumption in 13.5%. The most commonly used cessation method was cytisine (78.9%), followed by nicotine gum (10.9%), nicotine patch (4.3%), motivational interviewing (3.1%), varenicline (1.6%), and bupropion (1.2%). Awareness of secondhand smoke was high (84.4%), whereas awareness of thirdhand smoke was remarkably low (4.5%).

The distributions of Fagerström nicotine dependence level, smoking cessation status, and withdrawal symptoms across psychological dependence categories are presented in Table 2. Chi-square tests of independence revealed statistically significant associations between psychological dependence level and Fagerström nicotine dependence level (χ^2^ = 69.6, df = 6, *p* < 0.001), smoking cessation status (χ^2^ = 55.1, df = 4, *p* < 0.001), and withdrawal symptoms (χ^2^ = 6.11, df = 2, *p* = 0.047). Post hoc analyses employing standardized residuals revealed distinct patterns of association. Regarding Fagerström nicotine dependence level, in the low psychological dependence group, low and very low nicotine dependence were observed more frequently than expected (26.4% and 7.3%, standardized residuals = +4.384 and +4.189, respectively), while high nicotine dependence was observed less frequently than expected (23.6%, standardized residual = −4.426). In the moderate psychological dependence group, moderate nicotine dependence was observed more frequently than expected (47.3%, standardized residual = +2.578). In the severe psychological dependence group, high nicotine dependence was observed more frequently than expected (65.1%, standardized residual = +6.010), while moderate and low nicotine dependence were observed less frequently than expected (30.2% and 4.8%, standardized residuals = −3.130 and −3.340, respectively). Regarding smoking cessation status, in the low psychological dependence group, those who quit smoking were observed more frequently than expected (60.0%, standardized residual = +5.47), while those who did not quit were observed less frequently than expected (22.7%, standardized residual = −4.08). In the moderate psychological dependence group, those who reduced smoking were observed more frequently than expected (28.2%, standardized residual = +3.130). In the severe psychological dependence group, those who did not quit were observed more frequently than expected (61.9%, standardized residual = +5.90), while those who quit (22.2%, standardized residual = −4.11) and those who reduced smoking (15.9%, standardized residual = −2.14) were observed less frequently than expected. Regarding withdrawal symptoms, in the low psychological dependence group, the presence of withdrawal symptoms was observed less frequently than expected (41.8%, standardized residual = −2.01), while the absence of withdrawal symptoms was observed more frequently than expected (58.2%, standardized residual = +2.01). In the severe psychological dependence group, the presence of withdrawal symptoms was observed more frequently than expected (57.9%, standardized residual = +1.98), while the absence of withdrawal symptoms was observed less frequently than expected (42.1%, standardized residual = −1.98). No significant deviations were observed in the moderate psychological dependence group.

A Pearson correlation analysis revealed a moderate, positive, and significant relationship between psychological dependence and nicotine dependence (r = 0.41, *p* < 0.001). As psychological dependence increases, the severity of nicotine dependence also increases. The coefficient of determination (r^2^ = 0.168) suggests that approximately 16.8% of the variance in nicotine dependence is explained by psychological dependence.

Psychological dependence scores were compared across sociodemographic characteristics and smoking-related variables. The analyses revealed statistically significant differences according to gender, smoking cessation status, and withdrawal symptoms. When psychological dependence scores were examined according to smoking cessation status, participants who could not quit smoking had significantly higher psychological dependence scores (M = 54.0, SD = 9.46) compared to those who quit (M = 46.8, SD = 9.01) and those who reduced their smoking (M = 50.8, SD = 8.79). Participants who quit smoking had the lowest psychological dependence scores. The effect size was large (η^2^ = 0.167), indicating that smoking cessation status explains approximately 16.7% of the variance in psychological dependence scores. Regarding gender, female participants scored significantly higher on psychological dependence (M = 51.6, SD = 9.35) than male participants (M = 49.8, SD = 9.80), with a small effect size (Cohen’s d = 0.19). This result suggests that women may have stronger psychological reliance on nicotine compared to men. Regarding withdrawal symptoms, participants who experienced withdrawal symptoms had significantly higher psychological dependence scores (M = 51.3, SD = 9.63) than those who did not (M = 49.8, SD = 9.65) (*p* = 0.040). No statistically significant differences were found in psychological dependence scores according to alcohol use (*p* = 0.051), presence of chronic disease (*p* = 0.507), medication use (*p* = 0.667), knowledge of secondhand smoke (*p* = 0.666), knowledge of thirdhand smoke (*p* = 0.411), marital status (*p* = 0.407), education level (*p* = 0.446), or income level (*p* = 0.622). Although alcohol use showed a trend toward higher psychological dependence scores among those who consume alcohol (M = 52.4 vs. 50.3), this difference did not reach statistical significance (Table 3).

A multiple linear regression analysis was conducted to determine nicotine dependence based on the variables of psychological dependence, age, and pack-years; the overall model was statistically significant (F(3, 509) = 56.2; *p* < 0.001) and explained 24.9% of the variance in nicotine dependence (R^2^ = 0.249, Adjusted R^2^ = 0.245). The model summary is presented in Table 4. Psychological dependence showed the strongest association with nicotine dependence (β = 0.375, *p* < 0.001), followed by pack-years (β = 0.299, *p* < 0.001). For each one-unit increase in psychological dependence score, nicotine dependence increased by 0.080 units. Similarly, each additional pack-year was associated with a 0.028-unit increase in nicotine dependence. Age was not significantly associated with nicotine dependence (β = −0.027, *p* = 0.539) (Table 4).

Estimated marginal means of nicotine dependence were calculated at three levels of psychological dependence: one standard deviation below the mean (40.9), the mean (50.5), and one standard deviation above the mean (60.2). As shown in Figure 2, nicotine dependence scores increased progressively across psychological dependence levels. At one standard deviation below the mean psychological dependence score, the estimated marginal mean of nicotine dependence was 6.03 (SE = 0.113, 95% CI: 5.81–6.25). At the mean psychological dependence level, the estimated marginal mean was 6.81 (SE = 0.079, 95% CI: 6.65–6.96). At one standard deviation above the mean, the estimated marginal mean increased to 7.58 (SE = 0.113, 95% CI: 7.36–7.80). These findings further support the positive linear relationship between psychological dependence and nicotine dependence, demonstrating that higher psychological dependence scores are associated with substantially higher predicted nicotine dependence levels (Figure 2).

To determine smoking cessation status, a multinomial logistic regression analysis was conducted using the “failure to quit” category as the reference group. The model included gender, nicotine dependence, psychological dependence, education level, and income level as predictor variables. Although the model was found to be statistically significant (χ^2^(14) = 75.4; *p* < 0.001), the Nagelkerke R^2^ was 0.094, indicating that the model explained 9.4% of the variance in smoking cessation status. Psychological dependence showed the strongest association when comparing “quitters” and “non-quitters”. It has been observed that the probability of quitting smoking decreases by 8% for every one-unit increase in the psychological dependence score (OR = 0.920; 95% CI: 0.896–0.944; *p* < 0.001). In this comparison, no other variables including gender (OR = 1.253; *p* = 0.320), nicotine dependence (OR = 0.973; *p* = 0.640), education (high school vs. below high school: OR = 1.273; *p* = 0.497; university vs. below high school: OR = 1.758; *p* = 0.108), or income (income equal to expenses vs. income less than expenses: OR = 1.418; *p* = 0.259; income greater than expenses vs. income less than expenses: OR = 1.768; *p* = 0.109) reached statistical significance. In the comparison between those who reduced their smoking and those who did not, psychological dependence was again the only variable significantly associated. Each one-unit increase in the psychological dependence score was associated with a 2.9% decrease in the likelihood of reducing cigarette consumption (OR = 0.971; 95% CI: 0.944–0.998; *p* = 0.034). None of the other predictors such as gender (OR = 1.214; *p* = 0.430), nicotine dependence (OR = 0.925; *p* = 0.221), education level, or income level showed a significant association with the reduction in cigarette consumption (Table 5).

## 4. Discussion

This study aimed to examine the relationships between psychological dependence level and nicotine dependence, smoking cessation status, and withdrawal symptoms in individuals presenting to a smoking cessation outpatient clinic, as well as to determine the predictors of these processes. The most striking finding of our study is that psychological dependence was the factor most strongly associated with nicotine dependence and with a significantly lower likelihood of smoking cessation. These results demonstrate that smoking dependence is not merely a physiological process, but that psychological components are at least as determinant as physiological components. In our study, a moderate, positive, and significant correlation was found between psychological dependence and nicotine dependence (r = 0.41, *p* < 0.001). Furthermore, multiple linear regression analysis identified psychological dependence as the variable most strongly associated with nicotine dependence (β = 0.375, *p* < 0.001). This finding emphasizes that smoking dependence should be addressed within the framework of a biopsychosocial model. It suggests that in addition to the physically addictive effect of nicotine, the meanings attributed by individuals to the act of smoking also play an important role in maintaining dependence. In a study conducted by Aslan et al. among medical students, a moderate positive correlation (r = 0.459) was found between physical dependence and psychological dependence. This indicates that as physical dependence increases, psychological dependence tends to increase as well. No significant difference was found between sexes in terms of psychological dependence. Students with a “poor” socioeconomic status had higher psychological dependence levels compared to those with moderate and good socioeconomic status. It is emphasized that smoking cessation interventions should involve a holistic approach targeting both physical and psychological components [11].

Vijayakarthikeyan et al. found high levels of physical nicotine dependence in 44.1% of tobacco users and moderate levels in 29.6%. Of the participants, 13% had high, 28.5% had moderate, and 31.3% had mild psychological dependence, while 27.2% of users had no psychological dependence. Male sex, initiation of tobacco use before age 30, and use for 10 years or longer were found to be associated with high physical dependence and psychological dependence [12]. The moderate level of the relationship between physical and psychological dependence suggests that nicotine dependence cannot be explained solely by psychological factors, and that physiological and genetic factors are also involved in the process. Nevertheless, the fact that psychological dependence explains approximately 16.8% of the variance in nicotine dependence (r^2^ = 0.168) reveals the importance of psychological assessment in clinical practice. One of the findings of our study is that as the level of psychological dependence increases, smoking cessation success decreases significantly. In the low psychological dependence group, the rate of those who quit smoking was significantly higher than expected (60.0%), while in the severe psychological dependence group, the rate of those who could not quit was significantly higher than expected (61.9%). Multinomial logistic regression analysis supported this finding; a one-unit increase in psychological dependence score was found to decrease the likelihood of quitting smoking by 0.920-fold (OR = 0.920, 95% CI: 0.896–0.944). These findings suggest that psychological dependence may be a more determining factor than physical dependence in the smoking cessation process. Kutlu et al. found that smoking cessation success among individuals with high psychological dependence was significantly lower compared to those with low dependence. The study found that the mean psychological dependence score was 14.18 among those who quit smoking, while it was 15.70 among those who could not quit. Participants classified in the severe dependence category according to the TAPDS test were unable to quit smoking at the end of the first month. A significant positive correlation was found between psychological dependence and physical nicotine dependence (FTND) as well as the urge to smoke. It was observed that as psychological dependence increased, physical dependence also increased. The study emphasized that in smoking cessation clinics, not only physical dependence but also psychological dependence must be assessed, and that focusing on coping strategies for the urge to smoke during the treatment process would increase success [13]. de Granda-Orive et al. emphasized that treatment adherence, fewer previous failed attempts, and not viewing smoking as a psychological relaxation tool play critical roles in long-term success. Individuals who used smoking less as a calming tool were found to have a higher chance of quitting [14]. In our study, psychological dependence was also found to significantly decrease the likelihood of reducing smoking (OR = 0.971, *p* = 0.034). This indicates that psychological dependence negatively affects not only complete cessation but also partial successes such as reduction in quantity. This suggests that individuals with high psychological dependence have lower flexibility in smoking behavior and that intervention strategies should be specifically tailored to this group.

Our study revealed that psychological involvement also exacerbates withdrawal symptoms; withdrawal symptoms were less pronounced when psychological issues were minimal, whereas they were observed to be present in cases of high psychological severity. This finding suggests that psychological dependence may affect the perceived severity and experience of withdrawal symptoms. In the study conducted by Khosravani et al., positive metacognitions about smoking were found to be significant predictors of withdrawal symptoms, independent of other strong factors such as psychological distress and urge to smoke. This finding demonstrates that individuals’ thoughts and beliefs about the act of smoking directly influence how they experience withdrawal symptoms. The authors emphasized that addressing such beliefs during treatment should be an important therapeutic goal [15]. Withdrawal is not merely a biological substance deficiency; it is a complex process that emerges through the combination of genetic factors, environmental triggers, and highly routinized behavioral patterns. Highly routinized behavioral patterns refer to rituals and habits deeply embedded in daily life regarding the act of smoking. These routines represent not only the individual’s physical dependence on nicotine but also the cognitive and behavioral connections established with the act of smoking. These routines are automatically triggered by internal or external stimuli through Pavlovian associations or classical conditioning. When an individual quits smoking, the inability to perform these established routines leads to “behavioral withdrawal” symptoms. This situation produces symptoms similar to pharmacological withdrawal, such as craving, irritability, frustration, and concentration loss. Resisting these routinized responses may deplete the individual’s cognitive resources and self-regulation capacity, leading to “cessation fatigue.” Research has shown that even smoking denicotinized cigarettes can reduce withdrawal symptoms, demonstrating that the smoking ritual itself can activate reward systems in the brain independently of the drug. Highly routinized behavioral patterns define when, where, and in what emotional state smoking becomes an automatic response and may be the underlying cause of prolonged cravings that persist even after the biological withdrawal process (2–4 weeks) has ended [16].

In the comparison of psychological dependence scores according to sociodemographic characteristics, female participants were found to have significantly higher psychological dependence scores than males (*p* = 0.016). Research has shown that women use smoking more than men for mood control and to reduce negative affectivity (anxiety, anger, fear) during high arousal states. They tend to use nicotine as a coping mechanism in negative emotional states [17]. Therefore, in smoking cessation interventions for women, strengthening psychosocial support and emotion regulation skills should be prioritized. In the multiple linear regression analysis, in addition to psychological dependence, the pack-year variable was also significantly associated with nicotine dependence (β = 0.299, *p* < 0.001), while age was not a significant predictor. The association between pack-years and nicotine dependence indicates that the amount of nicotine exposure is related to the level of physiological dependence. However, the fact that psychological dependence is a stronger predictor (β = 0.375 vs. 0.299) demonstrates that, in addition to physical exposure, psychological processes also play a critical role in determining the severity of dependence. Dijkstra et al. examined the extent to which the FTND, originally developed to measure physical dependence, also reflects psychological tobacco dependence. Psychological dependence indicators (expected withdrawal symptoms, self-efficacy, etc.) explained approximately 20% of the variance in FTND scores. Nicotine dependence is both at physiological and psychological levels, with pack year being related to dose–response relationship which indicates both intensity and duration. In multivariate linear regression analysis, the pack-year variable was found to be significantly associated with nicotine dependence (FTND) score [18]. In our study, multinomial logistic regression analysis demonstrated that psychological dependence was significantly associated with smoking cessation status. A one-unit increase in psychological dependence score was associated with lower odds of quitting smoking (OR = 0.920, 95% CI: 0.896–0.944, *p* < 0.001) and of reducing smoking (OR = 0.971, 95% CI: 0.944–0.998, *p* = 0.034). This finding suggests that psychological dependence may be a more determining factor than physical dependence in the smoking cessation process.

Although studies directly examining the effect of psychological dependence on smoking cessation success are limited in the literature, available evidence supports this relationship. Research has shown that psychological dependence is an important component of nicotine dependence and may play a stronger role than physical dependence in maintaining smoking behavior [19]. In particular, the association of smoking with psychological functions such as emotional regulation, stress management, social acceptance, and image makes it difficult for individuals to quit smoking unless they develop alternative mechanisms to fulfill these functions [20]. The mechanisms underlying the negative effect of psychological dependence on smoking cessation are various. First, individuals with high psychological dependence tend to use smoking as a tool to cope with negative emotional states [6]. When they quit smoking, they experience withdrawal symptoms more intensely because they lose this emotional regulation tool, and the risk of relapse increases. Second, smoking behavior becomes associated with various environmental stimuli over time (e.g., drinking coffee, driving, social environments), and these conditioned stimuli trigger strong urges to smoke during the cessation process [21]. Automatic responses given when exposed to these stimuli may suppress cognitive control efforts and lead to unsuccessful quit attempts. Furthermore, the finding that psychological dependence significantly decreases the likelihood of reducing smoking (OR = 0.971) is noteworthy. This finding indicates that psychological dependence negatively affects not only complete cessation but also partial successes such as reducing quantity. This suggests that individuals with high psychological dependence have lower flexibility in smoking behavior and may have an "all or nothing" smoking pattern. This finding of our study emphasizes that psychological dependence should be routinely assessed in smoking cessation treatment and that intervention strategies should be developed accordingly. In particular, in treatment protocols for individuals with high psychological dependence, it is recommended that psychosocial interventions such as cognitive behavioral therapy, motivational interviewing, stress management, and emotion regulation skills training be integrated into pharmacological treatment [22]. This multidisciplinary approach may increase treatment success by helping individuals develop alternative coping strategies that can replace smoking. In practical terms, routine assessment of psychological dependence could be readily embedded into existing cessation services by administering a brief TAPDS screening at the initial visit and using the resulting score to stratify patients, so that individuals with high psychological dependence are flagged for stepped, adjunctive psychosocial support alongside standard pharmacotherapy. Within such a stepped-care model, cognitive behavioral therapy could target cue reactivity and automatic smoking routines; motivational interviewing could strengthen quit motivation and resolve ambivalence; stress-management and relaxation techniques could provide alternatives to smoking for affect regulation; and emotion-regulation training could help patients tolerate negative emotional states without smoking. These components could be delivered by trained clinic staff or an on-site psychologist, with intensity matched to the patient’s psychological dependence level. From a theoretical standpoint, these findings extend biopsychosocial and reinforcement-based models of tobacco dependence by showing that psychological dependence, operationalized through a standardized multidimensional instrument, contributes to cessation outcomes over and above physical nicotine dependence assessed with the FTND, thereby positioning psychological dependence as an independent and clinically actionable construct rather than a mere by-product of physical dependence.

### Strengths and Limitations

This study has several strengths. First, the prospective cohort design allowed for longitudinal follow-up of the smoking cessation process. Second, the large sample size included in the study (*n* = 513) increased the power of the statistical analyses. Third, the TAPDS, used to assess psychological dependence, is a multidimensional measurement tool with established Turkish validity and reliability studies. However, our study also has some limitations: (1) The study was conducted at a single center (Malatya Training and Research Hospital), which may limit the generalizability of the results. Replication of similar studies in smoking cessation clinics in different geographical regions is recommended. (2) The fact that participants were already presenting to a smoking cessation clinic indicates that they are a motivated group regarding quitting. Therefore, the results cannot be directly generalized to the general smoking population. As the study was conducted in a single clinic with a treatment-seeking and motivated sample, the associations reported here should be regarded as specific to this clinical population, and multicenter studies in community and primary-care settings, including individuals not actively seeking treatment, are needed before the findings can be generalized more broadly. (3) Smoking cessation status and withdrawal symptoms were largely based on participants’ self-reports; this may lead to social desirability bias or recall bias. In future studies, objective assessment could be performed using biochemical verification (e.g., carbon monoxide or cotinine measurement). (4) Treatment methods were not randomized but determined by the clinician, which may lead to confounding factors in treatment selection (e.g., disease severity, patient preference). (5) Although psychiatric disorders such as depression and anxiety that could affect psychological dependence levels were excluded, subclinical psychological problems were not assessed. (6) The TAPDS was analyzed at the total-score level; although it is a multidimensional instrument (Emotional Regulator, Image/Self-Reward, Indifference, and Social Acceptance), subdimension-level relationships with smoking cessation outcomes were beyond the scope of the present analysis and represent an important direction for future research. (7) Cytisine was the most frequently used pharmacotherapy in our sample (78.9%); because cytisine is not universally available and is not established as a first-line treatment in many Western countries, this heavy reliance on cytisine may limit the direct generalizability of the treatment-related findings to settings where nicotine replacement therapy, varenicline, or bupropion predominate. (8) Although smoking cessation status was assessed prospectively, the relationship between psychological dependence and nicotine dependence was examined cross-sectionally; therefore, the observational design does not allow definitive causal inferences, and the reported associations should be interpreted with caution.

## 5. Conclusions

According to the results of our study, psychological dependence is strongly associated with nicotine dependence and with lower smoking cessation success. Withdrawal symptoms are more frequently observed in individuals with high psychological dependence, and this complicates the cessation process. Female sex was found to be associated with higher psychological dependence. In smoking cessation treatment, the assessment of psychological dependence and the integration of psychosocial interventions into treatment with a multidisciplinary approach are of critical importance in increasing treatment success. Future studies should focus on a more detailed examination of the subdimensions of psychological dependence and the mediating mechanisms in this process. In particular, future studies should test whether psychological dependence mediates the relationships between sociodemographic, behavioral, and clinical factors and smoking cessation outcomes, examine the predictive value of the individual TAPDS subdimensions, and incorporate objective, biochemically verified cessation endpoints (e.g., carbon monoxide or cotinine measurement) across diverse populations and settings.

## Figures and Tables

**Figure 1:**
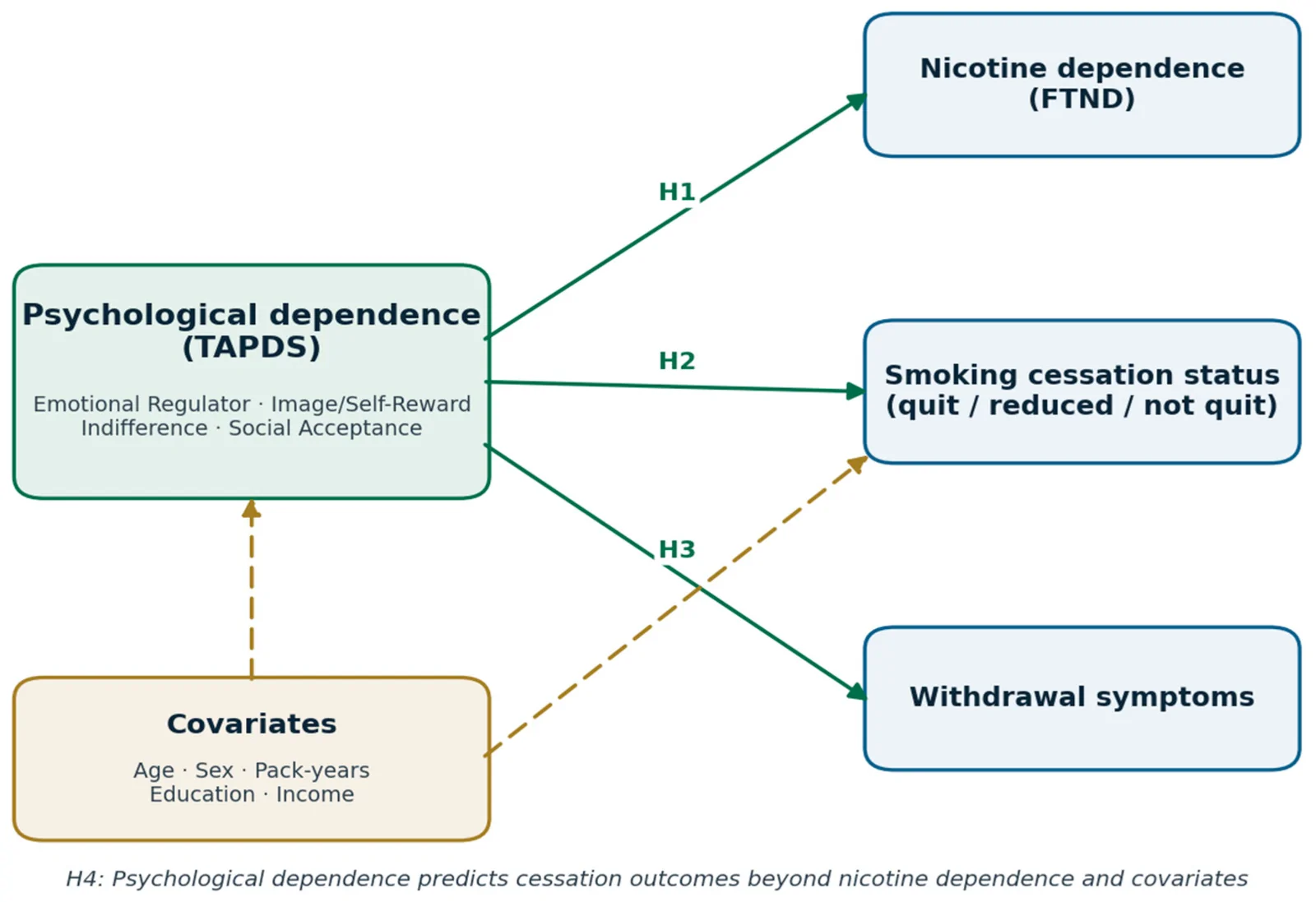
Conceptual framework of the study. Psychological dependence, assessed with the multidimensional Test to Assess the Psychological Dependence on Smoking (TAPDS), is examined as the focal predictor of nicotine dependence, smoking cessation status, and withdrawal symptoms, with sociodemographic and smoking-related characteristics considered as covariates.

**Figure 2:**
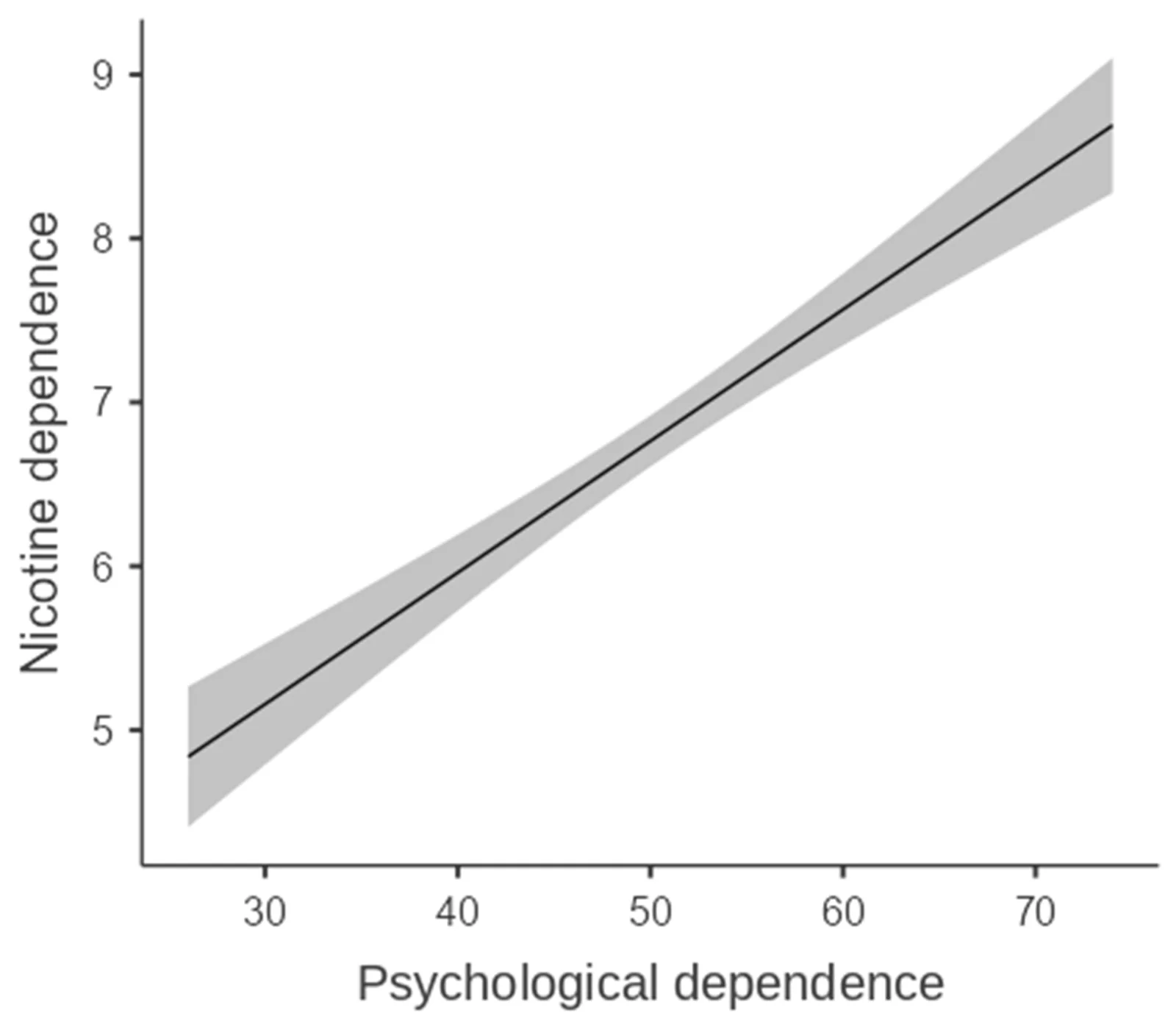
Estimated marginal means of nicotine dependence by psychological dependence level. Data are presented as estimated marginal means with 95% confidence intervals. The shaded band around the line represents these 95% confidence intervals. Psychological dependence levels are shown as mean − 1 SD (40.9), mean (50.5), and mean + 1 SD (60.2). A significant positive linear trend was observed across psychological dependence levels, with nicotine dependence scores increasing progressively from 6.03 to 7.58 (*p* < 0.001). Estimated marginal means were derived from the regression model adjusting for age and pack-years (*n* = 513).

**Table 1:** Demographic and clinical characteristics of participants.

Variable	Category	*n* (%)
Age (years)	Mean ± SD (Range)	36.7 ± 10.6 (18.0–65.0)
Pack-years	Mean ± SD (Range)	24.7 ± 21.7 (1.0–150.0)
Sex	Female	203 (39.6)
	Male	310 (60.4)
Marital status	Married	306 (59.6)
	Single	177 (34.5)
	Divorced	30 (5.8)
Education level	Below high school	68 (13.3)
	High school	177 (34.5)
	University	268 (52.2)
Employment status	Housewife	108 (21.1)
	Retired	30 (5.8)
	Employed	321 (62.6)
	Student	21 (4.1)
	Unemployed	33 (6.4)
Income level	Income < expenses	95 (18.5)
	Income = expenses	281 (54.8)
	Income > expenses	137 (26.7)
Comorbidity	Present	116 (22.6)
	Absent	397 (77.4)
Medication use	Present	107 (20.9)
	Absent	406 (79.1)
Alcohol use	Present	69 (13.5)
	Absent	444 (86.5)
Treatment method	Cytisine	405 (78.9)
	Nicotine gum	56 (10.9)
	Nicotine patch	22 (4.3)
	Motivational interviewing	16 (3.1)
	Varenicline	8 (1.6)
	Bupropion	6 (1.2)
Awareness of secondhand smoke	Yes	433 (84.4)
	No	80 (15.6)
Awareness of thirdhand smoke	Yes	23 (4.5)
	No	490 (95.5)

Values are presented as *n* (%) for categorical variables and as mean ± SD (range) for continuous variables. SD: standard deviation.

**Table 2:** Distribution of Fagerström dependence level, smoking cessation status and withdrawal symptoms by psychological dependence level.

		Psychological Dependence Level			
	Total (*n*)	Low *n* (%)	Moderate *n* (%)	Severe *n* (%)	*p*
Fagerström Dependence Level					
High	216	26 (23.6) [−4.426]	108 (39.0) [−1.549]	82 (65.1) [+6.010]	<0.001
Moderate	216	47 (42.7) [+0.149]	131 (47.3) [+2.578]	38 (30.2) [−3.130]	
Low	70	29 (26.4) [+4.384]	35 (12.6) [−0.722]	6 (4.8) [−3.340]	
Very Low	11	8 (7.3) [+4.189]	3 (1.1) [−1.798]	0 (0.0) [−1.910]	
Smoking Cessation Status					
Quit	193	66 (60.0) [+5.47]	99 (35.7) [−0.953]	28 (22.2) [−4.11]	<0.001
Not Quit	203	25 (22.7) [−4.08]	100 (36.1) [−1.741]	78 (61.9) [+5.90]	
Reduced	117	19 (17.3) [−1.56]	78 (28.2) [+3.130]	20 (15.9) [−2.14]	
Withdrawal Symptoms					
Present	258	46 (41.8) [−2.01]	139 (50.2) [−0.055]	73 (57.9) [+1.98]	0.047
Absent	255	64 (58.2) [+2.01]	138 (49.8) [+0.055]	53 (42.1) [−1.98]	
Total	513	110 (100.0)	277 (100.0)	126 (100.0)	

Data are presented as *n* (column %) [standardized residual]. Standardized residuals with |residual| > 1.96 indicate significant deviation from expected frequencies.

**Table 3:** Comparison of psychological dependence scores across groups.

	Variable/Group	M	SD	*p*	Effect Size
Sex	Female	51.6	9.35	0.016	0.19
	Male	49.8	9.80		
Comorbidity	Yes	50.6	9.71	0.507	0.01
	No	50.5	9.65		
Alcohol use	Yes	52.4	9.47	0.051	0.22
	No	50.3	9.66		
Medication use	Yes	50.3	9.83	0.667	0.03
	No	50.6	9.62		
Knowledge of secondhand smoke	Knows	50.5	9.82	0.666	0.03
	Does not know	50.8	8.78		
Knowledge of thirdhand smoke	Knows	50.9	8.50	0.411	0.04
	Does not know	50.5	9.71		
Marital status	Married	50.7	9.48	0.407	0.010
	Single	50.7	9.92		
	Divorced	48.2	9.92		
Education level	Below high school	51.0	9.61	0.446	0.0087
	High school	49.8	9.51		
	University	50.9	9.77		
Income level	Income < expenses	50.7	10.27	0.622	0.0043
	Income = expenses	50.8	9.39		
	Income > expenses	49.9	9.80		
Smoking cessation status	Quit	46.8	9.01	<0.001	0.167
	Could not quit	54.0	9.46		
	Reduced	50.8	8.79		
Withdrawal	Yes	51.3	9.63	0.040	0.16
	No	49.8	9.65		

M: mean; SD: standard deviation. Effect sizes are Cohen’s d for two-group comparisons and η^2^ for comparisons across three or more groups.

**Table 4:** Multiple linear regression for factors associated with nicotine dependence.

Predictor	Estimate	SE	t	*p*	Stand. Estimate	95% CI Lower	95% CI Upper	VIF	Tolerance
Intercept	2.241	0.515	4.349	<0.001	-	-	-	-	-
Psychological dependence	0.080	0.008	9.680	<0.001	0.375	0.299	0.451	1.02	0.982
Age	−0.005	0.009	−0.615	0.539	−0.027	−0.114	0.060	1.33	0.753
Pack-years	0.028	0.004	6.695	<0.001	0.299	0.211	0.386	1.35	0.742

SE: standard error; CI: confidence interval; VIF: variance inflation factor.

**Table 5:** Multinomial logistic regression analysis for smoking cessation status (reference: not quit).

Predictor	Estimate	SE	Z	*p*	Odds Ratio	95% CI Lower	95% CI Upper
Quit vs. Not Quit							
Intercept	3.496	0.718	4.867	**<0.001**	32.979	8.069	134.786
Gender (Male vs. Female)	0.225	0.226	0.995	0.320	1.253	0.804	1.952
Nicotine dependence	−0.027	0.059	−0.468	0.640	0.973	0.867	1.092
Psychological dependence	−0.084	0.013	−6.343	**<0.001**	0.920	0.896	0.944
Education (Ref: Below high school)							
High school	0.241	0.356	0.679	0.497	1.273	0.634	2.555
University	0.564	0.352	1.606	0.108	1.758	0.883	3.502
Income (Ref: Less than expenses)							
Equal to expenses	0.349	0.310	1.128	0.259	1.418	0.773	2.603
Greater than expenses	0.570	0.356	1.602	0.109	1.768	0.880	3.551
Reduced vs. Not Quit							
Intercept	1.149	0.787	1.460	0.144	3.156	0.674	14.765
Gender (Male vs. Female)	0.194	0.246	0.789	0.430	1.214	0.750	1.965
Nicotine dependence	−0.078	0.064	−1.223	0.221	0.925	0.817	1.048
Psychological dependence	−0.030	0.014	−2.125	**0.034**	0.971	0.944	0.998
Education (Ref: Below high school)							
High school	0.567	0.388	1.460	0.144	1.762	0.824	3.770
University	0.572	0.390	1.465	0.143	1.771	0.825	3.806
Income (Ref: Less than expenses)							
Equal to expenses	−0.214	0.306	−0.700	0.484	0.807	0.443	1.470
Greater than expenses	−0.349	0.375	−0.929	0.353	0.706	0.338	1.473

SE: standard error; CI: confidence interval. Values in bold indicate statistical significance (*p* < 0.05).

## Data Availability

The data presented in this study are available on request from the corresponding author. The data are not publicly available due to privacy restrictions relating to the participants of the smoking cessation outpatient clinic.

## Abbreviations

The following abbreviations are used in this manuscript:

ANOVA	Analysis of variance
CFA	Confirmatory factor analysis
CFI	Comparative fit index
CI	Confidence interval
FTND	Fagerström Test for Nicotine Dependence
IQR	Interquartile range
NFI	Normed fit index
OR	Odds ratio
RMSEA	Root mean square error of approximation
SD	Standard deviation
SE	Standard error
TAPDS	Test to Assess the Psychological Dependence on Smoking
TLI	Tucker–Lewis index
VIF	Variance inflation factor
